# Influence of contraceptive use and other socio-demographic factors on under-five child mortality in Bangladesh: semi-parametric and parametric approaches

**DOI:** 10.1186/s40834-023-00217-z

**Published:** 2023-03-03

**Authors:** Golam Rabbi Khan, Abdul Baten, Md. Abul Kalam Azad

**Affiliations:** Department of Statistics, Jagannath University, Dhaka-1100, Bangladesh

**Keywords:** Under-five mortality, Bangladesh, Contraceptive use, Socio-demographic, Cox PH model, Weibull model

## Abstract

**Background:**

The under-five child mortality rate is a widely accepted indicator of the development of a country as well as reflects the country’s health care system and quality of life. Although the child mortality rate is decreasing over time in Bangladesh, the rate is still high among South Asian countries. The target of the Sustainable Development Goal-3.2 is to reduce the under-five mortality rate in all countries of the world to 25 or fewer per 1000 live births by 2030. The purpose of this study is to identify the socio-demographic factors which have an influence on under-five child mortality in Bangladesh as well as to examine whether contraceptive use has any effect on under-five mortality in Bangladesh. Finally, a comparison has been made between the results obtained from the Cox proportional Hazard Model and Weibull model to find out which model is more efficient for the study data.

**Methods:**

For the study, data was extracted from Bangladesh Demographic Health Surveys 2017–2018 (BDHS 2017–2018). The Kaplan–Meier survival function has been used to demonstrate the survival probabilities of under-five children. While multivariate analyses of the Cox Proportional Hazard model and Weibull model are used to estimate the under-five mortality risks for various predictors.

**Results:**

The study results show consistently higher survival probabilities for children of mothers who used modern contraceptives during survival periods. Other significant predictors for under-five child mortality include mother’s education level (higher education), mother’s age (> 20), wealth index (rich), source of drinking water (tube well), and division (Chittagong, Khulna, Mymensingh). Weibull model has given more efficient results than the Cox Proportional Hazard model except for one covariate (water source).

**Conclusion:**

Contraceptives use significantly improves the survival chances of children under-five age. This underscores the importance of contraceptive use in the pursuit of a sustainable reduction in under-five mortality in Bangladesh. It also intensifies the need to address the present level of contraceptive use in the country. This may not be due to the use of contraceptives in itself but may be due to the substantial biological and socioeconomic benefits that are concomitant with contraceptive use which may promote both maternal and child health. So, Extra effort should be given by the policymakers to ensure the use of modern contraceptive methods to improve the under-five survival in Bangladesh.

**Supplementary Information:**

The online version contains supplementary material available at 10.1186/s40834-023-00217-z.

## Background

The under-five child mortality rate is a widely accepted indicator of the development of a country. It is also crucial evidence of a country’s values and priorities as well as reflects the country’s health care system and quality of life [1, 2]. Under-five child mortality rate refers to the probability of a child dying between birth and exactly five years of age, expressed per 1000 live births [3]. In 2020, 5 million children under five years of age died. That means that 13,800 children under the age of five were dying every day in 2020 [3].

To prevent child deaths and ensure healthy survival reduction of under-five mortality rate to 25 or below by 2030 was set as Sustainable Development Goal (SDG)-3.2 [1]. The global under-five mortality rate declined by 61 percent, from 93 deaths per 1000 live births in 1990 to 37 in 2020 [3]. Despite this considerable progress, improving child survival remains a matter of urgent concern.

Although the child mortality rate is decreasing over time, the rate is still high among South Asian countries [4]. Bangladesh is a lower-middle-income country in South Asia with a high under-five child mortality rate. The under-five child mortality rate has declined gradually in Bangladesh over the last 2 decades. Recently the country achieved a significant reduction in under-five mortality following the successful implementation of the MDG [1]. Infant and neonatal mortality rates remained stable in Bangladesh during the last few years and it is about 38 and 30 deaths per 1,000 live births respectively [5]. There is no doubt that extensive work and continued efforts are important for ensuring further reduction of under-five mortality to achieve the respective SDG targets. So the study on under-five child mortality is an important and contemporary public health issue in Bangladesh. With the growing emphasis on the implementation of family planning progress in recent times, finding out the determinants of child mortality and its trends is also getting important [6]. Family planning has been found to significantly contribute to the prevention of maternal and child mortality [6]. Globally, birth spacing through the increased use of modern family planning methods has been found to save the lives of more than two million newborns and children every year [7]. Contraception can also improve perinatal outcomes and child survival, mainly by lengthening inter pregnancy intervals. In developing countries, the risk of prematurity and low birth weight doubles when conception occurs within 6 months of a previous birth, and children born within 2 years of an elder sibling are 60% more likely to die in infancy than are those born more than 2 years after their sibling [8]. Improved access to family planning could also be a primary preventative measure to reduce under-five deaths. Despite the progress made in ensuring access to modern contraceptive methods in recent times, women have continued to report on the unmet need for family planning in developing countries [9]. The unmet need for contraception is high in most parts of the developing world as a result of low contraceptive use [10, 11].

Worldwide several studies have been undertaken that focused on the socio-economic determinants of infant and child mortality. For instance, in Kenya, Muriithi and Muriithi employed Cox regression survival analysis to the Kenya Demographic and Health Survey data for children to determine the effect of socio-economic and demographic factors on infant and child mortality [12]. Nasejje et al. examined the determinants of under-five mortality in Uganda by employing Cox proportional hazard model with frailty effects under both the frequentists and Bayesian approaches [13]. A multivariate cox regression analysis has been performed by Niser and Dibley to determine the potential risk factors of neonatal mortality in Pakistan [14]. Like other developing countries, in Bangladesh, social scientists and policymakers are also greatly interested in the factors affecting under five-mortality to accelerated socio-economic development and improved quality of life [1]. Karmakar et al. examined the determinants of under-five mortality in Bangladesh using the cox proportional hazard model to BDHS data [15]. While Islam et al. determined that socio-economic and demographic factors are significantly associated with child mortality by running a logistic regression model [16]. Cox regression analysis has been also performed by Chowdhury and Rahman to identify the factors affecting child survival in Bangladesh [17, 18].

While many studies have examined the determinants of under-five mortality in Bangladesh, there is a gap in knowledge on whether contraceptive use and intention to use have any implications for under-five mortality in Bangladesh. The research documented that contraceptive use has the potential to improve perinatal outcomes and child survival by widening the interval between successive pregnancies [8]. It has been found that the risk of death in ages 1–4 years would fall by 21 percent in developing countries if all children were spaced by at least two years age gap [8]. In a study, it was found that the risk of under-five mortality is higher for children whose mothers had an unmet need for family planning than those whose mothers had met the need for family planning [19]. Similarly, family planning is found to be a major factor in reducing the overall rate of under-five mortality in Bangladesh [2]. Moreover, evidence shows that birth interval is an index of contraceptive use, and has implications for childhood mortality [20–22].

Therefore the key interest of this paper is to examine the effect of contraceptive use and intention to use on under-five child mortality in Bangladesh. The study also controlled other factors that may confound the effect of contraceptive use and intention to use on under-five mortality in Bangladesh. Finally, we compared the results of the Cox proportional hazard model with the Weibull model to measure the efficiency of the models.

## Methods

### Sources of data

The analysis is based on secondary data obtained from BDHS 2017–18 which was conducted through a collaborative effort of the National Institute of Population Research and Training (NIPORT), ICF International, USA, and Mitra & Associates. We have collected this BDHS 2017–18 data from the Demographic Health Survey (DHS) after following their rules and regulations. Detailed information about sampling methodologies and data collection procedures can be found in the BDHS reports [23].

### Sample design & size

The BDHS 2017–2018 used a sampling frame from the list of enumeration areas (EAs) of the 2011 population and housing census of the People’s Republic of Bangladesh that was conducted by the Bangladesh Bureau of Statistics (BBS). The primary sampling unit (PSU) for the survey was considered an EA that was consisted of an average of about 120 households. These surveys were based on a two-stage stratified sample of households. In the first stage, 675 EAs were selected using the probability proportional to the EA size, with 425 EAs in rural areas and 250 in urban areas respectively and in the second stage of sampling, a systematic sample of 30 households on average was selected per EA to provide statistically reliable estimates of key demographic and health areas separately for each of the divisions. The details of the sample design can be found in the BDHS 2017–2018 Bangladesh Demographic and Health Survey (BDHS) report [23]. Since in this study, data was restricted to children under age 5. Based on these criteria, the sample size for this study was 8759 children that were extracted from BDHS 2017–2018 data.

### Variables

#### Outcome variable

The dependent variable in this study is time to death for children under the age of five years. This time is recorded in months for our analysis. That is the outcome variable is the survival time in months of the children under the age of five years. We have observed children up to five years of their age. If a child dies before its fifth birthday then an event occurs and if a child is alive on its fifth birthday then it is considered censored.

#### Explanatory variable

In this study, the explanatory variables are categorized into two factors socioeconomic factors and demographic factors. On the one hand, socio-economic factors include the Mothers’ education, Mothers’ working status, Place of residence, Wealth index, Access to media, Division, Religion, and Water source variables. On the other hand, demographic factors include Contraceptive use, Mothers’ age, the Sex of child, and Birth order variables. Selection of these explanatory variables were based on the review of the existing studies examined the factors influencing the under-five mortality in Bangladesh [14–18]. In BDHS surveys, use of contraception is defined as the proportion of currently married women who report using a family planning method at the time of the survey [5]. In the data set contraceptive use variable was given as categorical variable with five categories which were using modern method, Using traditional method, Non-user—intends to use later, Does not intend to use, Never had sex. For the study purpose these five categories were converted into three categories. The first category was remain same as a second category and third category was also remain same as a third category but the Using traditional method, Does not intend to use, Never had sex categories jointly constitute the first category Non User. The Mother’s education variable was given as with four categories like No Education, Primary, Secondary and Higher. We converted this variable into three categories like the first category was remain same as a first category and fourth category was also remain same as a third category but the second and third categories considered jointly as a second category. The Media Variable was categories as, if mothers were reading newspaper or magazine/listening to radio/watching television, then ‘Yes’ category is used for them. However, ‘No’ category was used, when mothers were not associated with any of the above activities.

### Statistical analyses

First of all, to assess the association between the dependent and explanatory variables, we used the product-limit estimation and Log-rank test procedure since the dependent variable is time-related. After that to identify the risk factors, we used the Cox proportional hazard model [24] (semi-parametric) and the Weibull model (parametric). In this study, two software are used; one is SPSS and another is R programing. Finally, we measured the efficiency of the Cox proportional hazard model and Weibull model by comparing the relative efficiency of the results from both models. The relative efficiency was measured by using this formula:

$$RE=\frac{Standard\,error\,of\,the\,parameter\,of\,Weibull\,Model}{Standerd\,error\,of\,the\,parameter\,of\,Cox\,PH\,Model}$$

### Ethical considerations

The study used secondary data from the DHS database under the rules and regulations of the databases. As a one of the host country of the DHS, when BDHS was conducted a written consent obtained from all the respondents before each interview was conducted. Most importantly, the informed written consent statement emphasizes that the respondent’s identity and information will be kept strictly confidential [25]. All procedures and questionnaires of standard DHS surveys have been reviewed and approved by Institutional Review Board (IRB), and ICF international. Moreover, country-specific DHS survey protocols are reviewed by the IRB, ICF international, and generally by an IRB in the host country (DHS, 2022) [25].

## Results

### Descriptive results

From Table 1, we observe that among the mothers who were interviewed, 10% of mothers were the non-user of contraceptive methods, 61.3% had used modern methods and 28.7% had non-user but intend to use them. Among the mothers 7.3% had no education, 76.1% had completed primary & secondary education and 16.6% had completed higher education. Of the mother who gave the interview, 12.4% of them were aged between 15 and 19 years of age and 87.6% were between 20 and 49. We also observed that among all the mothers, 59.3% of mothers had no working status and 40.7% had a working status. Of the study participants 34.9% were from urban and 65.1% from rural areas. Among the mothers, 42% of mother’s socioeconomic status were low, 17.8% of mother’s socioeconomic status were middle, and 40.1% of mother’s socioeconomic status were high. We also see that among 8 divisions, the highest proportion of mothers live in Chittagong and which is about 16.5% whereas the lowest portion in Khulna it’s near about 10.3%. Among the mothers, 91.5% were Muslim and 8.5% were from other religions. It was also observed that among the children, 52.1% were male and 47.9% were female. It is found that 38.6% of the children were the first baby of their mothers and 61.4% baby’s birth order is other. And 63.9% of mothers were exposed to media while 36.1% were not. It is noticeable that 6.3% of women used piped water as drinking water, 79.6% tube well water, and 14.2% others.

**Table 1:** Percentage and frequency distribution of the selected socio-economic and demographic variables, BDHS 2017–2018

**Variable**	**Category**	**Frequency**	**Percentage**
Contraceptive Use	Non user	880	10.0
	Modern method	5365	61.3
	Non user but intend to use	2514	28.7
Mother’s Education	No	642	7.3
	Primary & Secondary	6663	76.1
	Higher	1454	16.6
Mother’s Age	15–19	1084	12.4
	20–49	7675	87.6
Mother’s Working Status	No	5195	59.3
	Yes	3564	40.7
Place of Residence	Urban	3057	34.9
	Rural	5702	65.1
Wealth quintile	Low	3683	42.0
	Middle	1563	17.8
	High	3513	40.1
Division	Barisal	906	10.4
	Chittagong	1446	16.5
	Dhaka	1304	14.9
	Khulna	904	10.3
	Mymensingh	1025	11.7
	Rajshahi	912	10.4
	Rangpur	971	11.1
	Sylhet	1291	14.7
Religion	Muslim	8018	91.5
	Others	741	8.5
Sex of Child	Male	4567	52.1
	Female	4192	47.9
Birth Order	First birth	3383	38.6
	Others	5376	61.4
Access to Media	No	3158	36.1
	Yes	5601	63.9
Water Source	Piped	549	6.3
	Tube Well	6970	79.6
	Others	1240	14.2

### Contraceptive use and under-five survival

**Fig. 1:**
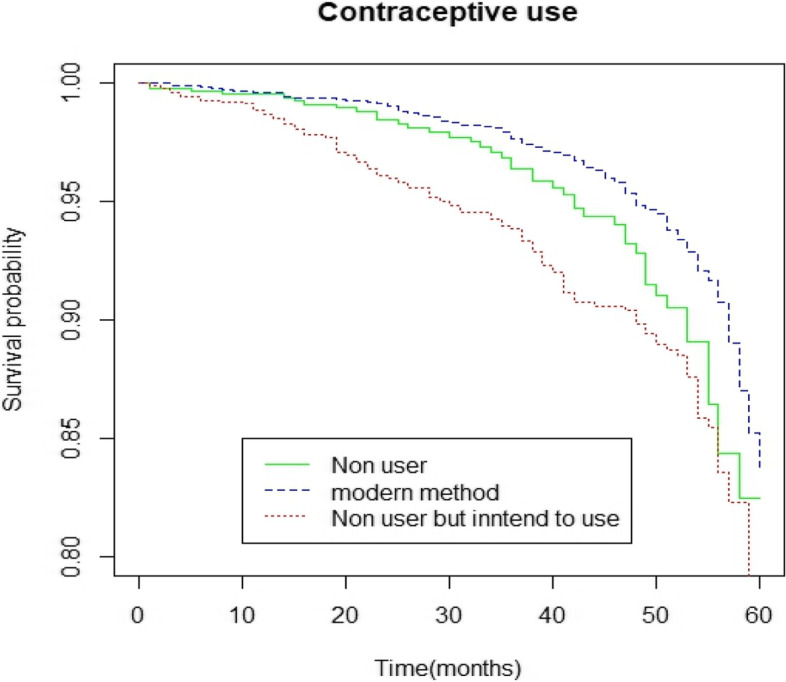
Survival curves for use of the Contraceptive method (Log-Rank test: *p*-value < 0.0001)

The variable Contraceptive use is categorized into three groups: Non-user, Use modern method, and Non-user but intend to use. Here we determine the relationship between child survival probability and contraceptive use throughout the survival (0–59 months) period. To show this relationship of survival probability these three levels are estimated by the P-L estimator and the Log-Rank test is conducted to obtain the *p*-value (Fig. 1). In the figure, the survival curves for different categories of contraceptive use are shown along with the log-rank test *p*-value. From the diagram, it is observed that the children born of mothers who were use modern method were at a greater chance of surviving to the age of five years than children born of mothers who were not use and intend to use. To examine the highly significant difference among different groups of contraceptive use, log-rank test is performed and obtained the *p*-value. Since the *p*-value is (*p* < 0.0001) the survival experience among different groups differ significantly. That is why contraceptive use is potential determinant for under-5 child mortality.

### Contraceptive use and other risk factors for under-five children mortality

We observed that both unadjusted models (Cox PH model and Weibull model) give almost similar results (Table 2). Hazard ratios are approximately equal in both models. Confidence intervals for the Cox PH model are also approximately similar to the Weibull model. From Tables 2 and 3, we observed that there is a little difference between the results of adjusted and unadjusted models (Weibull and Cox PH model) for our main variable of interest ‘use of contraceptives’. So, the other covariates might be the risk-factors for under-five mortality.

**Table 2:** Estimates of parameters for under-five mortality using Cox Proportional Hazard Model (Unadjusted) and Weibull Model (Unadjusted)

**Model**	**Variable**	**HR**	**SE(**${\varvec{\beta}})$	**95%CI**	***p*-value**
Cox	Contraceptive:	-	-	-	-
Proportional	Non user (Ref:)				
Hazard Model (Unadjusted)	Modern method	0.6713	0.1682	[0.4829, 0.9333]	0.0010***
	Non-user but intend to use	1.4653	0.1715	[1.0492, 2.0472]	0.0250*
Weibull	Contraceptive:	-	-	-	-
Model (Unadjusted)	Non user (Ref:)				
	Modern method	0.6657	0.1680	[0.4788, 0.9255]	0.0010***
	Non-user but intend to use	1.4654	0.1705	[1.0491, 2.0469]	0.0261*

NB: Ref = Reference Category *p*• ≤ 0.1; p* ≤ 0.05; p**≤ 0.01; p^***^≤ 0.001

**Table 3:** Estimates of parameters for under-5 mortality using Cox Proportional Hazard Model & Weibull Model (Adjusted)

Variables	HR & 95%CI (Cox PH)	HR & 95%CI (Weibull)	*P*-value (Cox PH)	***P***-value (Weibull)	Cox PH(RE)
**Contraceptive use:**					
Non user (Ref:)	-	-	-	-	-
Modern method	0.659 [0.473, 0.919]	0.657 [0.470, 0.910]	0.0010***	0.0010***	0.9978
Non-user but intend to use	1.502 [1.069, 2.109]	1.524 [1.085, 2.141]	0.0188*	0.0160*	0.9985
**Mother’s education level:**					
No education (Ref:)	-	-	-	-	-
Primary/secondary	0.928 [0.642, 1.340]	0.913 [0.632, 1.319]	0.6887	0.6270	0.9985
Higher	0.613 [0.364, 1.033]	0.602 [0.358, 1.014]	0.0658^•^	0.0580^•^	0.9957
**Mother’s age:**					
15–19 (Ref:)	-	-	-	-	-
20–49	0.283 [0.204, 0.395]	0.302 [0.217, 0.419]	< 0.0001***	< 0.0001***	0.9927
**Media:**					
No (Ref:)	-	-	-	-	-
Yes	0.962 [0.748, 1.238]	0.973 [0.757, 1.251]	0.7653	0.8310	0.9954
**Wealth quintile:**					
Low (Ref:)	-	-	-	-	-
Middle	1.021 [0.752, 1.385]	1.016 [0.749, 1.378]	0.8964	0.9190	0.9974
High	0.795 [0.659, 1.213]	0.793 [0.659, 1.209]	0.0674^•^	0.0670^•^	0.9957
**Residence:**					
Rural (Ref:)	-	-	-	-	-
Urban	0.879 [0.614, 1.015]	0.894 [0.618, 1.021]	0.6049	0.7030	0.9955
**Working status:**					
No (Ref:)	-	-	-	-	-
Yes	1.035 [0.826, 1.297]	1.054 [0.840, 1.321]	0.7658	0.6510	0.9931
**Religion:**					
Islam (Ref:)	-	-	-	-	-
Others	1.360 [0.957, 1.933]	1.373 [0.967, 1.949]	0.8061	0.7070	0.9982
**Birth order:**					
First birth (Ref:)	-	-	-	-	-
Others	1.209 [0.944, 1.551]	1.215 [0.948, 1.558]	0.1334	0.124	0.9996
**Sex of child:**					
Male (Ref:)	-	-	-	-	-
Female	0.846 [0.686, 1.044]	0.862 [0.699, 1.063]	0.1182	0.1650	0.9972
**Water sources:**					
Piped (Ref:)	-	-	-	-	-
Tube well	0.742 [0.619, 1.654]	0.734 [0.608, 1.624]	0.0409*	0.0421*	1.0002
Others	0.705 [0.692, 2.099]	0.702 [0.686, 2.079]	0.0419*	0.0431*	0.9991
**Division:**					
Barisal (Ref:)	-	-	-	-	-
Chittagong	0.705 [0.465, 1.067]	0.697 [0.461, 1.055]	0.0984^•^	0.0890^•^	0.9974
Dhaka	0.891 [0.582, 1.363]	0.878 [0.575, 1.345]	0.5936	0.5520	0.9992
Khulna	0.708 [0.442, 1.132]	0.718 [0.449, 1.149]	0.0149*	0.0168*	0.9993
Mymensingh	0.668 [0.423, 1.054]	0.684 [0.434, 1.079]	0.0831^•^	0.1040^•^	0.9994
Rajshahi	0.917 [0.587, 1.435]	0.892 [0.570, 1.395]	0.7056	0.6150	0.9997
Rangpur	0.769 [0.489, 1.206]	0.782 [0.498, 1.226]	0.2526	0.2840	0.9987
Sylhet	0.953 [0.638, 1.422]	0.952 [0.638, 1.420]	0.8119	0.8090	0.9985

NB: Ref = Reference Category *p*• ≤ 0.1; p^*^ ≤ 0.05 ; p^**^ ≤ 0.01 ; p^***^ ≤ 0.001

From the unadjusted Cox PH and Weibull model (Table 2) it was found that the modern contraceptive method was statistically significant (*p* $\leq0.001$) for under-five child mortality in Bangladesh. From both unadjusted models we found that under-five children whose mothers used modern contraceptives were less likely (hazard ratio (HR) = 0.6713 and 0.6657, CI: [0.4829, 0.9333] and [0.4788, 0.9255] respectively) to die than those whose mothers did not use modern contraceptives.

Furthermore, the factor contraceptive use on under-five mortality was controlled for other socioeconomic and demographic factors of children and mothers in both Cox PH and Weibull models in order to determine the risk factors of under-five mortality in Bangladesh (Table 3). This is used to examine the association between contraceptive use and intention to use and child survival while adjusting for the effects of selected socio-economic and demographic factors on children and mothers. As shown in Cox PH and Weibull models (Table 3), after adjusting for the effects of selected socio-economic and demographic factors on children, the use of modern contraceptive methods remained highly significantly associated (*p* $\le 0.001$) with under-five mortality. From table-3 we have concluded that children whose mothers used modern contraceptive methods have a lower risk of dying (hazard ratio (HR) = 0.659 and 0.657, CI: [0.473, 0.919] and [0.470, 0.910] respectively) before reaching the age of five years than the children whose mothers not used modern contraceptive methods. At the same the Non-user but intend to use of modern contraceptive methods significantly associated (*p* < 0.016) with under-five mortality.

In both models at a 10% level of significance, higher education has a significant effect on under-five child mortality. Using both the Cox PH model and Weibull model, we observed that the children whose mothers have a higher education have a lower rate of mortality (hazard ratio (HR) = 0.613 and 0.602, CI: [0.364, 1.033] and [0.358, 1.014] respectively) than the children of the illiterate mother. Considering a 0.1% level of significance, the age group 20–49 has shown a highly significant impact on under-five mortality in both models. The children whose mother’s age are greater than 20 have a 71.7% lower risk of mortality in the Cox PH model than the children whose mother’s age is less than 20 whereas it is 69.8% in the Weibull model.

Using the Cox PH model and Weibull model we observed that the wealth index has a significant impact on under-five child mortality at a 10% level of significance. From both models, it was found that the children who are belonging to the high socioeconomic status have a lower rate of mortality (hazard ratio (HR) = 0.795 and 0.793, CI: [0.659, 1.213] and [0.659, 1.209] respectively) than the children who are belonging to low socioeconomic status. While the covariates Residence, media, working status, Religion and birth order have no significant effect on under-five mortality in both analyses. The sex of the child is not statistically significant in both models but female children have a lower risk of mortality than male children. From Table 3, we can conclude that water sources are significant at a 5% level of significance. From the Cox PH adjusted model, it was found that the children whose mothers drink water from the tube well and other freshwater sources have less mortality rate (hazard ratio (HR) = 0.742 and 0.734, CI: [0.619, 1.654] and [0.608, 1.624] respectively) than the children whose mother drink piped water. And from the Weibull model, we get a 25.8% & 26.6% lower risk of child mortality for mother who drinks tube well water and from other freshwater sources than the mothers who drink piped water. In Cox PH and Weibull model, the division Dhaka, Rajshahi, Rangpur and Sylhet are not statistically significant but Chittagong, Khulna and Mymensingh are statistically significant for under-five child mortality in Bangladesh at 10%, 5%, and 10% respectively.

Table 3 shows the relative efficiencies (RE) of Cox PH model parameters with respect to the Weibull model parameters. It is shown that all relative efficiencies are less than one for all variables except water sources. That is why we can say that for all variables except water sources the estimates obtained from the Cox PH model are less efficient compared to the Weibull model.

## Discussion

This study has examined the implication of contraceptive use and other socio-demographic factors which have an influence on under-five child mortality in Bangladesh. The data were based on live births among women who reported met needs or unmet needs for family planning during the period under study. In the study, first of all, survival functions between under-five mortality and contraceptive use are generated. The results reveal a higher childhood survival probability for children of mothers who used modern contraceptives than the children of mothers who were not willing to use contraceptives. After adjusting for the effects of important covariates such as maternal education, wealth status, marital status among others, water sources, and birth order in the multivariate analysis, the results still show a significant association between under-five mortality and contraceptive use. This finding implies that use of modern method is associated with lower under-five mortality in Bangladesh. A similar association is observed elsewhere in Ethiopia [26]. Contraception use can also improve perinatal outcomes and child survivals mainly by lengthening inter pregnancy intervals. The risk of premature baby and low birth weight doubles when conception occurs within 6 months of a previous birth [8]. This means that contraceptive use has a considerable impact on child survival in Bangladesh. This may not be due to the use of contraceptives in itself but may be due to the substantial biological and socioeconomic benefits that are concomitant with contraceptive use which may promote both maternal and child health [26].

The findings of this study suggest that higher education for females is needed since it contributes significantly to declining the rate of under-five mortality [1]. Mother’s education significantly associated with child survival, which contributes through different mechanisms. A high risk of child death among illiterate mothers compared with secondary or higher-educated mothers are also consistent with other study findings [27, 28]. Educated mothers have better socioeconomic status, good knowledge of family health and childcare, and are more conscious about child illness, preventive care, and effective use of modern health services [27–29]. In addition, education also helps mothers regarding decision-making and empowers them in various issues like childcare which in turn plays a role in reducing child mortality [30, 31].

Significant mortality differentials were observed by maternal age at the birth of the child. The findings reveal a higher risk of death for children of younger mothers which also confirms previous research findings [32, 33]. Maternal age at birth can influence child mortality from different perspectives. The higher risk of child death among younger mothers pertains because of immature reproductive systems and less stability to handle the complexities of childbirth [34]. Moreover, younger mothers are more likely to have low-birth-weight babies [35], which is associated with a higher risk of child death [36].

It has been found from the analysis that the wealth index has a significant effect on under-five mortality in Bangladesh and a child from a rich family has a lower risk of mortality than a poor family [1]. Several studies concluded that in Bangladesh children born to the family of women from poor wealth quintile have a higher under-five mortality than those born to women in the richest wealth quintile (HR: 0.64, CI 0.47–0.86, *p* < 0.01) [2, 37–39]. Further, results showed significantly a higher risk of death among children of mothers in poor households. It could be reasonably assumed that even if poor women know the contraceptive methods that are available for their use (either to limit or space births), they may be unaffordable to them. This is because contraceptive commodities are not free in most developing countries [40]. The issue of old-age security may be another strong reason for many poor people to desire to have so many children even if contraceptive commodities are available and affordable [40].

Lack of access to clean water has been considered to be one of the important factors that contribute to more than 80 percent of childhood deaths in the world [41], and this has been reflected in this study. The use of an unimproved source of drinking water is found to be associated with an increased risk of under-five child mortality. There is also considerable evidence from studies in developing countries that show the role of household sanitation and clean water supply in promoting child health and survival [42–44]. Thus, access to an improved source of water and sanitation may have positive effects on under-five mortality risks in Bangladesh [26].

Among the divisions Chittagong, Khulna and Mymensingh are statistically significant for child mortality. Among the divisions the Sylhet division showed significantly higher child mortality than the others. A significant decline in under-five mortality has also been observed over the years. High child mortality in the Khulna and Sylhet division was observed due to many factors, including religious influence, superstitions, and lower awareness about child and maternal health [45]. In another study, it was found that Khulna and Sylhet divisions have a significant effect on under-five mortality in Bangladesh [1]. A study also found that there is enormous variation in the child mortality rate across different regions in Bangladesh. For example, neonatal mortality was found to be higher in the Sylhet division, whilst both infant mortality and under-five mortality were found to be higher in the Sylhet division. A recent study conducted by Gruebner et al. found similar results [46].

Our study found that the Weibull model gives more efficient results than the Cox PH model for the analyzed data set. A study also found, when both models apply to a data set, the Cox model estimates are less accurate and less efficient than the Weibull model estimates [47].

The study has one notable limitation that should be taken with caution when interpreting the results. There is a possibility of a time difference between the occurrence of the outcome and the main explanatory variable (under-five mortality and contraceptive use), as contraceptive-using behavior of the mother may not always coincide with the occurrence of under-five mortality which may affect the results. However, we believe that this provides the best scenario of the phenomenon as we do not expect mothers’ contraceptive use behavior to change considerably within the five years preceding the survey. For this type of study, instead of DHS data, a follow-up study would have been a better alternative. In case of follow-up study, a cohort of women is followed up for five years, recording the death and birth histories of their children. Another limitation is BDHS data contained the collection of data from women age 15–49 who are alive in a given household but for mothers who have died, no information is collected. That’s why the results may be affected by some bias.

## Conclusion

The findings of this study show that contraceptive use and other socio-demographic factors have indispensable implications for under-five mortality risk in Bangladesh. The use of modern contraceptive methods significantly improves the survival chances of the age of under-five children. This underscores the importance of contraceptive use in the pursuit of a sustainable reduction in under-five mortality in Bangladesh. It also intensifies the need to address the present level of contraceptive use in the country. And the mother’s age has an important role in the under-five child mortality rate. To reduce this fact, we need to be concerned about early marriage in Bangladesh. And also need to be concern about female education levels because highly educated mother’s children have a lower mortality rate. Implementing a cost-effective public health-related intervention to improve household environmental conditions, such as access to improved sources of drinking water have a positive effect on reducing under-five mortality in the country. To improve under-five survival, the government of Bangladesh should take more necessary steps to increase the use of the contraceptive methods and should concern about other socio-demographic factors which have significant impact on under-five child mortality.

## Supplementary Information

**Additional file 1.**

## Data Availability

Secondary data from Bangladesh Demography and Health Survey has been used in the study [48].
